# Optimized and Validated Spectrophotometric Methods for the Determination of Enalapril Maleate in Commercial Dosage Forms

**DOI:** 10.4137/aci.s643

**Published:** 2008-03-01

**Authors:** Nafisur Rahman, Sk Manirul Haque

**Affiliations:** Department of Chemistry, Aligarh Muslim University, Aligarh (U.P.) 202 002, India.

**Keywords:** enalapril maleate, spectrophotometry, validation, commercial dosage forms

## Abstract

Four simple, rapid and sensitive spectrophotometric methods have been proposed for the determination of enalapril maleate in pharmaceutical formulations. The first method is based on the reaction of carboxylic acid group of enalapril maleate with a mixture of potassium iodate (KIO_3_) and iodide (KI) to form yellow colored product in aqueous medium at 25 ± 1°C. The reaction is followed spectrophotometrically by measuring the absorbance at 352 nm. The second, third and fourth methods are based on the charge transfer complexation reaction of the drug with p-chloranilic acid (pCA) in 1, 4-dioxan-methanol medium, 2, 3-dichloro 5, 6-dicyano 1, 4-benzoquinone (DDQ) in acetonitrile-1,4 dioxane medium and iodine in acetonitrile-dichloromethane medium. Under optimized experimental conditions, Beer’s law is obeyed in the concentration ranges of 2.5–50, 20–560, 5–75 and 10–200 μg mL^−1^, respectively. All the methods have been applied to the determination of enalapril maleate in pharmaceutical dosage forms. Results of analysis are validated statistically.

## Introduction

Enalapril maleate is chemically described as 1-[N-[(S) - 1 –carboxy- 3-phenylpropyl] L - proline 1’- ethyl ester, maleate (1:1). Its molecular weight is 492.53. Enalapril maleate is a prodrug; following oral administration, it is bioactivated by hydrolysis of ethyl ester to enalaprilat, which is the active angiotensin converting enzyme inhibitor. It lowers peripheral vascular resistance without causing an increase in the heart rate. It is an ideal drug for hypersensitive patients who are intolerant to beta-blockers. The drug is used for treating high blood pressure or hypertension in adults and children and congestive heart failure. The drug and their tablets are official in USP 24 (1), which describes HPLC method for its quantitation.

The therapeutic importance of enalapril maleate was behind the development of numerous methods for its determination. The methods adopted to the analysis of enalapril maleate include high-performance liquid chromatography (2–9), capillary electrophoresis (10), liquid chromatography- tandem mass spectrometry (11), polarography (12), atomic absorption spectrometry (13) and membrane selective electrodes (14).

Few spectrophotometric methods have also been reported for the determination of enalapril maleate in commercial dosage forms. The cited drug after dissolving in distilled water exhibits an absorbance maximum at 207 nm and this property has been exploited to develop a UV method for its quantitation (15). Enalapril maleate reacts with 2, 4-dinitroflurobenzene at pH 9 yielding a colored product which absorbs maximally at 356 nm forming a basis for its determination (12). Spectrophotometric methods based on the ternary complex formation between copper (II), eosin (13); palladium (II), eosin (16) and enalapril maleate have been reported. The spectrophotometric methods reported for analysis of enalapril maleate in commercial dosage forms suffered disadvantage of heating at 100 °C (12) and long analysis time (16). Considering this drawback, there was a need to develop more advantageous spectrophotometric methods for its determination in commercial dosage forms.

This paper describes four sensitive, fast, simple and economical methods for the determination of enalapril maleate in dosage forms. The first method is based on the reaction of carboxylic acid group of enalapril maleate with a mixture of potassium iodate and iodide. The second, third and fourth methods are based on the charge transfer complexation reaction of the drug with pCA in 1, 4-dioxan-methanol medium, DDQ in acetonitrile-1,4 dioxan medium and iodine in acetonitrile-dichloromethane medium

## Experimental

### Apparatus

Spectral runs were made on Spectronic 20D^+^ Spectrophotometer (Milton Roy Company, USA) with 1 cm matched glass cells.

### Materials and reagents

- Enalapril maleate was kindly provided by Sunij Pharma Pvt. Ltd., Ahmedabad, India and was used as received.
- Pharmaceutical preparations of enalapril maleate such as enapril (Intas Pharmaceuticals Pvt. Ltd., India), envas (Cadila Pharmaceuticals Ltd., India) and enace (Nicholas Primal India Ltd.) were purchased from local market.
- Potassium iodide (s.d. Fine-Chem Ltd, Mumbai, India) solution was prepared as 5.0 × 10^−2^ M solution in distilled water.
- Potassium iodate (Central Drug House (P) Ltd, New Delhi, India) solution was also prepared as 3.0 × 10^−3^ M solution in distilled water.
- pCA (Central Drug House (P) Ltd, New Delhi, India) was prepared as 0.2% (w/v) solution in 1,4-dioxan.
- DDQ (Fluka Chemie AG, Germany) was prepared as 0.1% (w/v) solution in 1, 4-dioxan.
- Iodine (Merck Limited, Mumbai, India) was prepared as 0.1% (w/v) solution in dichloro-methane.

### Standard drug solutions

Enalapril maleate standard solutions were prepared as 0.5 mg mL^−1^ solution in distilled water for Method A, 2.0 mg mL^−1^ in methanol for Method B, 0.5 mg mL^−1^ in acetonitrile for Method C and 1.0 mg mL^−1^ in acetonitrile for Method D.

### Procedure for determination

#### Method A

Aliquots of enalapril maleate standard solution (0.5 mg mL^−1^) containing 25–500 μg were transferred into a series of 10 mL volumetric flasks. To each flask, 1 mL of 3.0 × 10^−3^ M potassium iodate and 1.5 mL of 5.0 × 10^−2^ M potassium iodide were added and diluted to volume with distilled water. The reaction was allowed to proceed at 25 ± 1 °C and absorbance was measured as a function of time at 352 nm against reagent blank prepared simultaneously. The calibration curve was constructed by plotting the A (absorbance measured at 8.0 min—absorbance measured at 2 min) against the initial concentration of enalapril maleate. The content of enalapril maleate was calculated either from the calibration curve or corresponding regression equation.

#### Method B

Into a series of 5 mL volumetric flasks, volumes of enalapril maleate standard solution (2.0 mg mL^−1^) equivalent to 0.1–2.8 mg of the drug were transferred. To each flask, 2.5 mL of 0.2% pCA was added and brought up to the volume with 1,4 dioxan. The colored product formed immediately at room temperature (25 ± 1 °C) and absorbance was measured after 2 min of mixing at 510 nm against the reagent blank prepared similarly omitting the drug.

#### Method C

The aliquots of enalapril maleate standard solution (0.5 mg mL^−1^) equivalent to 25–375 μg of the drug were transferred into a series of 5 mL volumetric flasks. To each flask, 1.4 mL of 0.1% DDQ was added and brought up to the volume with acetonitrile. The color developed immediately at room temperature (25 ± 1 °C) and the absorbance was measured after 3 min at 565 nm against the reagent blank prepared similarly omitting the drug.

#### Method D

The volumes of enalapril maleate standard solution (1.0 mg mL^−1^) corresponding to 50–1000 μg of the drug were transferred into a series of 5 mL flasks. 1.2 mL of 0.1% iodine was added in each flask at room temperature (25 ± 1 °C) and diluted up to the mark with dichloromethane. The absorbance was measured after 2 min at 365 nm against the reagent blank.

### Analysis of pharmaceutical formulations

Twenty tablets (claming for 2.5 mg of enalapril maleate per tablet) were finely powdered and extracted separately into sufficient volume of water, methanol or acetonitrile with shaking. The residue was filtered on Whatmann filter paper No. 42 and the filtrate was diluted to 50 mL with water, methanol or acetonitrile, as the case may be. It was further diluted according to the need and then analyzed following the proposed procedures.

## Results and Discussion

### Reaction with a mixture of iodide and iodate

It has been reported in the literature (17) that iodine is formed as a result of the interaction of a mixture of iodide and iodate with inorganic or organic acid in accordance with the equation:

$$5I^{-}+10_{3}^{-}+6H^{+}\to 3H_{2}O+3I_{2}$$

**Scheme 1.**
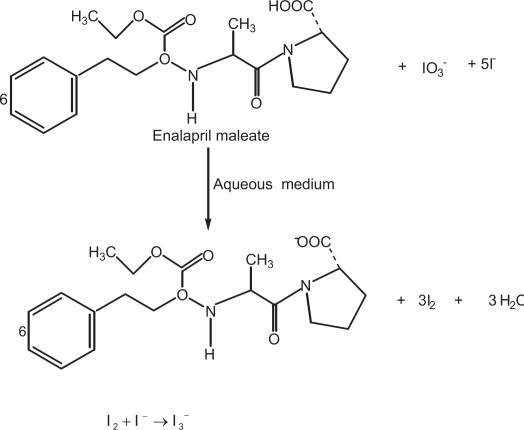


In aqueous solution, the iodide ions react with the liberated iodine to yield triiodide ion (*I*_2_+*I*^−^ → *I*_2_^−^) which absorbs maximally at 290 nm and 360 nm. We thought that this reaction would be helpful for developing a spectrophotometric method for determination of enalapril maleate as it contains –COOH group in its moiety. Keeping this in mind, a mixture of potassium iodide and potassium iodate was allowed to react with enalapril maleate which yielded iodine. Then the liberated iodine reacted with the excess of iodide ion resulting in the formation triiodide ion with λ_max_ at 352 nm. The reaction sequence is shown in Scheme 1.

### Reaction with σ-acceptor

**Figure 1.**
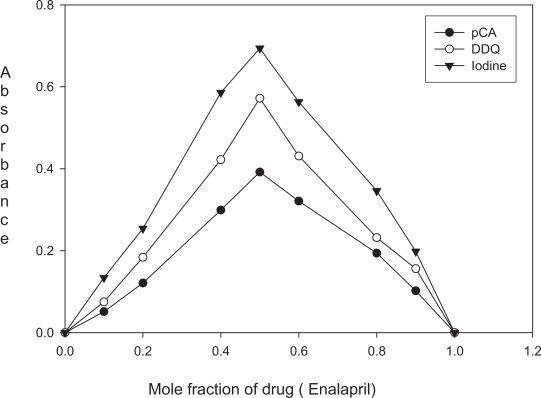
Job’s plot for stoichiometric ratio between enalaparil maleate and pCA (4.06 × 10^−3^ M each), DDQ (2.03 × 10^−3^ M each) or Iodine (3.94 × 10^−^^3^ M each).

**Scheme 2.**
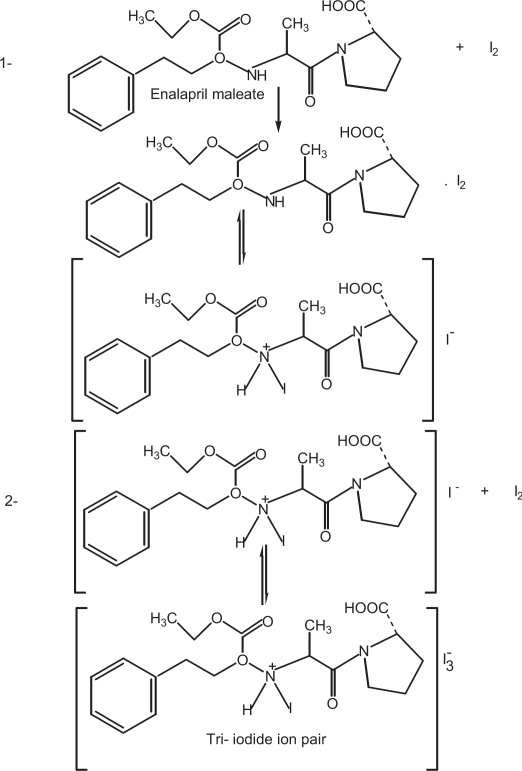


The absorption spectrum of iodine in dichloromethane showed only one peak with maximum absorption at 500 nm. The color of iodine changes to yellow upon reaction with enalapril maleate. This is due to charge transfer complexation reaction between enalapril maleate and iodine. The absorption spectrum of enalapril maleate-iodine reaction product showed absorption peaks at 290 and 365 nm. The stoichiometry of the reaction was studied by Job’s method of continuous variations. It was observed from the Figure 1 that the combining molar ratio between enalapril maleate and iodine is 1:1. It has been reported that the charge-transfer complex between drug and iodine (18) would have an ionized structure DI^+^ … I_3_^−^. The absorption spectrum of this charge-transfer complex is identical to that of I_3_^−^ in dichloromethane as it also absorbed at 290 nm and 360 nm. On the basis of our experimental findings and the literature background, the reaction mechanism is proposed and given in Scheme 2.

### Reaction with π-acceptors

The interaction of enalapril maleate with π-acceptors such as p-CA and DDQ at room temperature was found to yield colored charge transfer complexes. In polar solvents, complete electron transfer from enalapril maleate (D), as an electron donor, to the acceptor moiety (A) takes place resulting in the formation of intensely colored radical anions. The reaction sequence can be shown as:

$$\text{D}+\text{A}⇋\overset{\text{Polar solvent}}{\underset{\text{complex}}{\left(\text{D}-\text{A}\right)}⇋\underset{\text{radical ions}}{{\text{D}\cdot }^{+}+{\text{A}\cdot }^{-}}}$$

The absorption spectra of enalapril maleate- π-acceptor reaction mixtures showed absorption peaks which were similar to the maxima of the radical anions of the π-acceptors obtained by the iodide reduction method (19).

The literature reveals that pCA exists in three forms:(i) H^2^A at very low pH which is yellow-orange in color, (ii) HA^−^, dark purple, which is stable at pH 3, and (iii) colorless A^−2^ which is stable at high pH. The interconversion of these species can be represented as:

$$\begin{array}{l} {\text{H}}_{2}\text{A}⇌{\text{H}}^{+}+{\text{HA}}^{-}(\text{violet})\\ {\text{HA}}^{-}⇌{\text{H}}^{+}+{\text{A}}^{-}(\text{colorless}) \end{array}$$

**Scheme 3.**
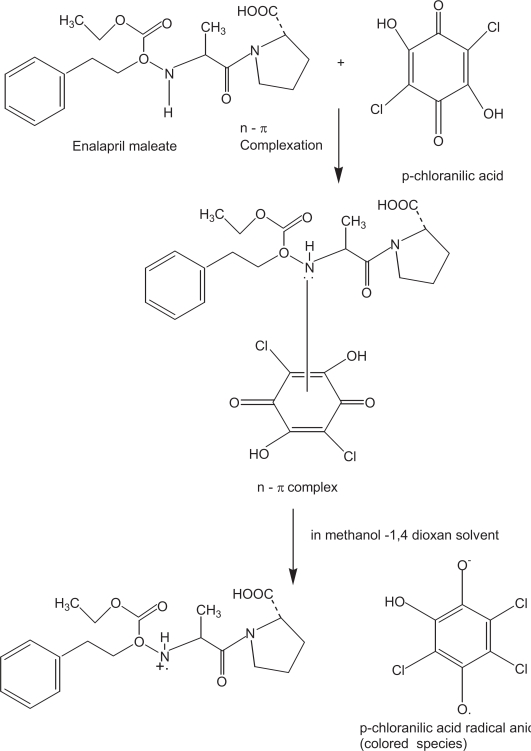


The interaction of enalapril maleate with pCA in methanol-1,4-dioxan solvent system resulted in the formation of violet colored charge transfer complex and the absorption spectrum exhibited maximum absorption at 510 nm. The reaction stoichiometry between enalapril maleate and pCA was evaluated by applying Job’s method of continuous variations. The Job’s plot (Fig. 1) reached a maximum value at a mole fraction of 0.5 which suggested a donor (enalapril maleate) to acceptor (pCA) ratio of 1:1. On the basis of reaction stiochiometry and literature background, the reaction mechanism is presented in scheme 3.

**Scheme 4.**
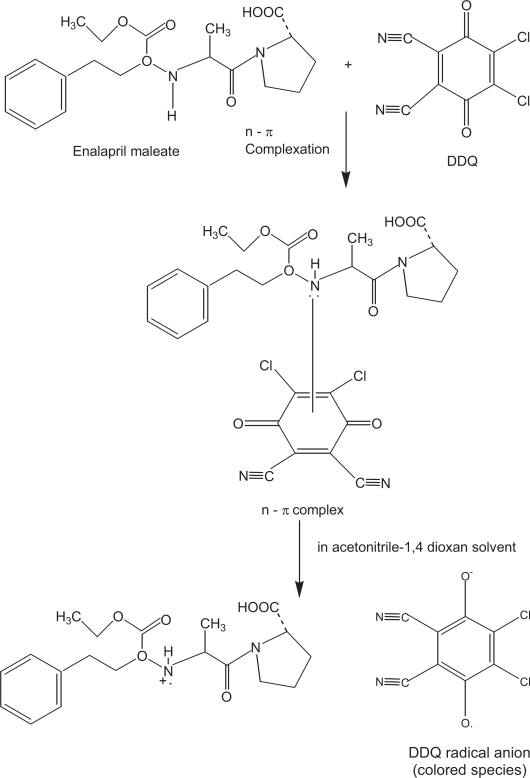


The interaction of enalapril maleate with DDQ resulted in the formation of colored charge-transfer complex. The dissociation of charge-transfer complex was promoted by the high ionizing power of the acetonotrile-1, 4-dioxan solvent system where complete electron transfer from enalapril maleate to the DDQ moiety takes place. This is followed by the formation of the DDQ radical anion as predominant chromogen which absorbed maximally at 430, 535 and 565 nm. In order to avoid the maximum interference from the blank, the absorption band at λ_max_ 565 nm was chosen for analytical studies. The Job’s plot (Fig. 1) indicated a donor to acceptor ratio of 1:1. On the basis of this study and literature survey, the formation of DDQ radical anion is shown in Scheme 4.

**Figure 2.**
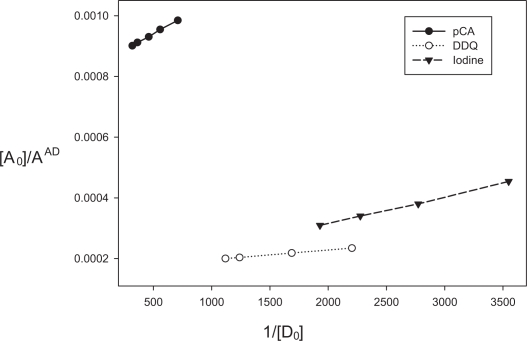
Plot of 1/[D_0_] vs [A_0_]/A^AD^ for Methods B, C and D.

The association constants and apparent molar absorptivities of pCA-enalapril maleate, DDQ-enalapril maleate and iodine-enalapril maleate charge transfer complexes have been calculated using Ross and Labes equation (20), which depends on the experimental condition that acceptor concentration should not low enough to be considered negligible with respect to donor concentration.

(1) $$\frac{\left[A][D\right]}{\left[A]+[D\right]}\times \frac{1}{A_{\lambda }}=\frac{1}{K\in_{\lambda }}\times \frac{1}{\left[A]+[D\right]}+\frac{1}{\in_{\lambda }}$$

where [*A*] and [*D*] are total concentrations of the acceptor and donor, respectively. A_λ_ and ɛ_λ_ are the absorbance and apparent molar absorptivity of the complex at wavelength λ. K is the association constant of the charge transfer complex. $\frac{\left[A][D\right]}{\left[A]+[D\right]}\times \frac{1}{A_{\lambda }}$ is plotted against $\frac{1}{\left[A]+[D\right]}$ (Fig. 2) which gave a straight line. The intercept and slope were calculated and equation (1) is transformed into the following equations:

for pCA – enalapril maleate system

(2) $$\begin{array}{ll} \frac{\left[A][D\right]}{\left[A]+[D\right]}\times \frac{1}{A_{\lambda }}= & 2.173\times 10^{-7}\times \frac{1}{\left[A]+[D\right]}\\ & +8.313\times 10^{-4} \end{array}$$

for DDQ – enalapril maleate system

(3) $$\begin{array}{ll} \frac{\left[A][D\right]}{\left[A]+[D\right]}\times \frac{1}{A_{\lambda }}= & 3.202\times 10^{-8}\times \frac{1}{\left[A]+[D\right]}\\ & +1.641\times 10^{-4} \end{array}$$

for Iodine – enalapril maleate system

(4) $$\begin{array}{ll} \frac{\left[A][D\right]}{\left[A]+[D\right]}\times \frac{1}{A_{\lambda }}= & 8.889\times 10^{-8}\times \frac{1}{\left[A]+[D\right]}\\ & +1.371\times 10^{-4} \end{array}$$

The association constants and molar absorptivities were found to be 3.826 × 10^3^ and 1.203 × 10^3^ Lmol^−1^cm^−1^ for pCA-enalapril maleate complex from equation (2), 5.124 × 10^3^ and 6.095 × 10^3^ Lmol^−1^cm^−1^ for DDQ-enalapril maleate complex from equation (3) and 1.543 × 10^3^ and 7.291 × 10^3^ Lmol^−1^cm^−1^ for iodine- enalapril maleate complex from equation (4), respectively. The free energy change was also calculated and found to be −20.44 KJmol^−1^ for pCA-enalapril maleate complex, −21.17 KJmol^−1^ for DDQ-enalapril maleate complex and −18.19 KJmol^−1^ for iodine- enalapril maleate complex.

### Optimizations of variables

The different parameters affecting the color development were extensively studied to determine the optimum conditions for the assay procedures. The optimum values of the variables were maintained throughout the determination process.

### Method A

#### Effect of the concentration of potassium iodate

**Figure 3.**
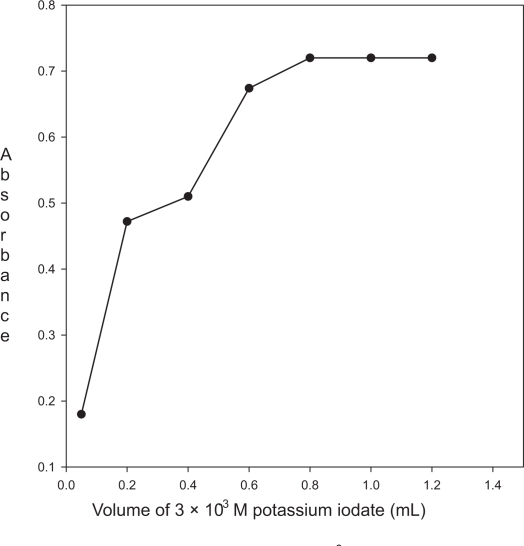
Effect of the volume of 3.0 × 10^−3^ M potassium iodate on the absorbance of the product (enalapril maleate 50 μg mL^−1^; 1.5 mL of 5.0 × 10^−2^ KI).

The effect of the volume of 3.0 × 10^−3^ M potassium iodate on the absorbance of the product was studied in the range of 0.2–1.2 mL. The absorbance increases with increase in the volume of KIO_3_ and became constant at 0.8 mL. Further addition of KIO_3_ does not change the absorbance and therefore, 1.0 mL of 3.0 × 10^−3^ M KIO_3_ was chosen as an optimum value (Fig. 3).

#### Effect of the concentration of potassium iodide

**Figure 4.**
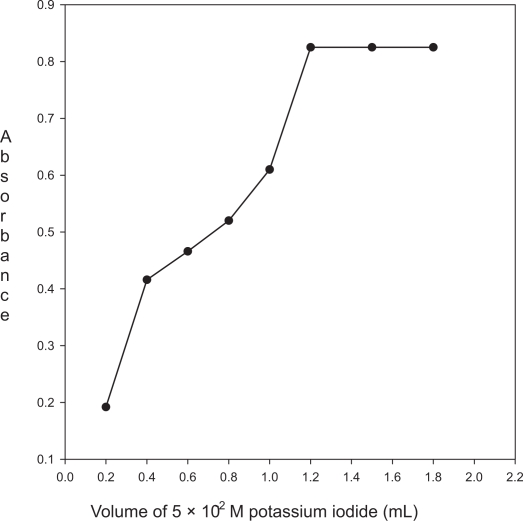
Effect of the volume of 5.0 × 10^−2^ M potassium iodide on the absorbance of the product (enalapril maleate 50 μg mL^−1^; 1.0 mL of 3.0 × 10^−3^ KIO_3_).

The effect of the volume of 5.0 × 10^−2^ M potassium iodide on the intensity of the colored product was studied in the range of 0.2– 1.8 mL, keeping the constant concentrations of enalapril maleate (50 μg mL^−1^) and KIO_3_ (3.0 × 10^−4^ M). The maximum absorbance was obtained with 1.2 mL; further addition caused no change on the absorbance. Thus, 1.5 mL of 5.0 × 10^−2^ M potassium iodide was used throughout the experiment (Fig. 4).

### Method B

#### Effect of reaction time

Optimum reaction time was evaluated by monitoring the color development at room temperature. It was observed that the reaction got stabilized within 2 min. The developed color remained stable at room temperature for about a further 1 h.

#### Effect of pCA concentration

**Figure 5.**
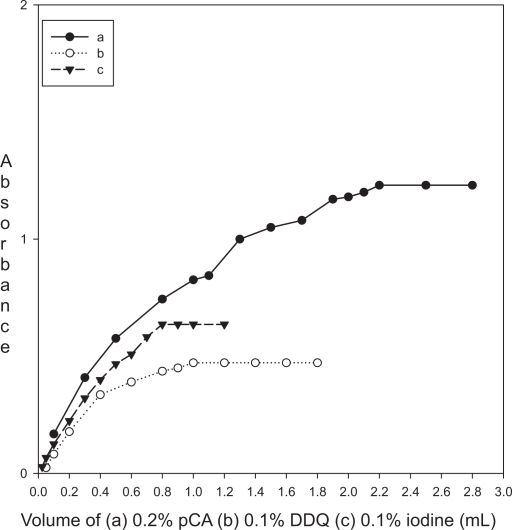
Effect of the volume of 0.2% pCA, 0.1% DDQ and 0.1% Iodine on the absorbance (600 μg mL^−1^ enalapril maleate for pCA, 50 μg mL^−1^ enalapril maleate for DDQ and 100 μg mL^−1^ enalapril maleate for iodine).

To study the effect of the volume of the reagent on the absorbance of the charge transfer complex, varying volumes of 0.2% pCA was mixed with 1.5 mL of 0.2% drug in a 5 mL standard flask and diluted to volume with 1, 4-dioxan. The results (Fig. 5) showed that the highest absorbance was obtained with 2.2 mL, which remained unaffected by further addition of pCA. Hence, 2.5 mL of the reagent was used for the determination.

### Method C

#### Effect of reaction time

The interaction of DDQ with enalapril maleate resulted in the formation of colored product which stabilized within 2 min. The developed color remained stable at room temperature for about a further 2 h.

#### Effect of DDQ concentration

To establish the optimum experimental condition, enalapril maleate (250 μg) was allowed to react with different volumes (0.05–1.8 mL) of 0.05% DDQ. The results (Fig. 5) showed that the highest absorbance was obtained with 1.0 mL, which remained unaffected by further addition of DDQ. Hence, a volume of 1.4 mL of the reagent was used for the determination.

### Method D

#### Effect of reaction time

In method D, the colored product was formed immediately and remained stable at room temperature for about 1 h. The absorbance was measured after 2 min. of mixing the reagent.

#### Effect of iodine concentration

In order to study the effect of the volume of 0.1% iodine on the absorbance of the charge transfer complex, varying volumes (0.05–1.2 mL) were treated, separately with 500 μg of enalapril maleate. The results are shown in Fig. 5 which indicated that 0.8 mL of 0.1% iodine gave the maximum absorbance and remained constant by further addition of iodine. Therefore, a volume of 1.2 mL was chosen as an optimum value for the determination.

#### Validation of proposed methods

Under the optimum experimental conditions, the calibration graphs were constructed by plotting Δ A (Δ A = absorbance measured at 8.0 min—absorbance measured at 2 min) or absorbance as a function of initial concentration of enalapril maleate for methods A; and B, C and D, respectively. The method of least square was applied to yield the regression equations. Results are summarized in Table 1. It is apparent from the table 1 that the sensitivity of the proposed methods follows the order: A>C>D>B. In all cases, calibration plots (n = 11) were linear with small intercepts (1.32 × 10^−4^ ± 6.52 × 10^−4^), (9.36 × 10^−6^ ± 2.43 × 10^−3^), (6.98 × 10^−4^ ± 1.55 × 10^−3^) and (−8.76 × 10^−4^ ± 4.12 × 10^−3^) and good correlation coefficients (0.9998–0.9999). The LOD values obtained for methods A, B, C and D were 1.13, 3.07, 0.39 and 3.20 μg mL^−1^ respectively. The small values of the variance 2.13 × 10^−7^, 3.03 × 10^−6^, 1.04 × 10^−6^ and 9.06 × 10^−6^ μg mL^−1^for methods A, B, C and D respectively (Table 1) confirmed the small degree of scatter of experimental data points around the line of regression.

### Accuracy and precision

The within day precision was evaluated through replicate analysis (n = 5) of quality control samples: 20, 35 and 50 μg mL^−1^ for method A and 120, 280 and 440 μg mL^−1^ for method B and 20, 40 and 60 μg mL^−1^ for method C and 40, 100 and 160 μg mL^−1^ for method D. The percentage recoveries ranged from 99.96–100.08% with RSD from 0.284%–0.845%, 99.96%–100.02% with RSD from 0.122%–0.242%, 99.96%–100.04% with RSD from 0.250%–0.481% and 100.01%–100.06% with RSD from 0.142%–0.440% for methods A, B, C and D respectively. The interday precision was also evaluated through replicate analysis of the quality control samples for five consecutive days at the same concentration levels as used in within day precision. The percentage recoveries for methods A, B, C and D ranged from 99.98%–100.11% with RSD from 0.482%–1.098%, 99.91%–99.96% with RSD from 0.184%–0.373%, 99.89%–100.00% with RSD from 0.373%–0.872% and 100.01%–100.06% with RSD from 0.318%–0.918% respectively (Table 2). The results in the Table 2 indicated the high precision of the proposed methods. The selectivity of the proposed methods was investigated by observing any interference encountered from the excipients of the tablets such as lactose, starch, cellulose, magnesium stearate, ferric oxide, talc and calcium carbonate and hydrochlorothiazide which is present with enalapril maleate in pharmaceutical products. It was observed that the excipients and hydrochlorothiazide did not interfere with the proposed methods.

The proposed methods were applied to the determination of enalapril maleate in commercial tablets such as Enapril 2.5, Envas 2.5 and Enace 2.5. The same batch of the commercial dosage forms was also analyzed by the reference method (12). The results of the proposed methods were compared with those obtained by the reference method. Statistical analysis of the results using Student’s t-test, variance ratio F-test and interval hypothesis test (21) revealed no significant difference between the proposed method (A, B, C and D) and reference method at 95% confidence level regarding accuracy and precision. The results are summarized in Table 3. The proposed methods have been extended to the determination of enalapril maleate in spiked human urine samples. The results of analysis of urine samples are summarized in Table 4.

## Conclusion

The proposed method does not require any laborious clean up procedure before measurement. In addition, the method has wider linear dynamic range with good accuracy and precision. The methods show no interference from the common excipients and additives. The statistical parameters and recovery data reveal the good accuracy and precision of the proposed methods. Therefore, it is concluded that the proposed methods are simple, sensitive and rapid for the determination of enalapril maleate in commercial dosage forms.

## Figures and Tables

**Table 1.** Optical characteristics and statistical data of the regression equations for methods A, B, C and D.

**Parameters**	**Method A**	**Method B**	**Method C**	**Method D**
**λmax (nm)**	352	510	565	365

**Beer’s law limit (μg mL^−1^)**	2.5–50	20.0–560	5–75	10–200
**Linear regression equation**	ΔA = 1.32 × 10^−4^+ 1.35 × 10^−3^ C	A = 9.36 × 10^−6^ + 1.87 × 10^−3^ C	A = 6.98 × 10^−4^+ 8.70 × 10^−3^ C	A = −8.76 × 10^−4^ + 3.11 × 10^−3^ C
**tS_a_**	6.15 × 10^−4^	2.30 × 10^−3^	1.46 × 10^−3^	3.88 × 10^−3^
**tS_b_**	2.11 × 10^−5^	6.37 × 10^−6^	3.13 × 10^−5^	6.53 × 10^−3^
**Correlation coefficient (r)**	0.9998	0.9999	0.9999	0.9999
**Variance (S_0_^2^)**	2.13 × 10^−7^	3.03 × 10^−6^	1.04 × 10^−6^	9.06 × 10^−6^
**Detection limit (μg mL^−1^)**	1.13	3.07	0.39	3.20

± t S, Confidence limit for intercept.

± t S, Confidence limit for slope.

**n** = 11, t (ΰ = 10) = 2.228

**Table 2.** Test of precision of the proposed methods A, B, C and D.

**Proposed methods**	**Concentration, (μg mL^−1^)**		**Recovery (%)**	**RSD^a^ (%)**	**SAE^b^**	**CL^c^**
	**Taken**	**Found ± SD**				
**Method A**						
Intraday assay	20	19.99 ± 0.169	99.96	0.845	0.076	0.210
	35	35.03 ± 0.133	100.08	0.378	0.059	0.165
	50	50.01 ± 0.142	100.03	0.284	0.063	0.176
Interday assay	20	20.02 ± 0.220	100.11	1.098	0.098	0.273
	35	35.00 ± 0.169	100.00	0.482	0.076	0.210
	50	49.99 ± 0.270	99.98	0.538	0.120	0.334
**Method B**						
Intraday assay	120	119.99 ± 0.290	99.99	0.242	0.130	0.360
	280	279.89 ± 0.478	99.96	0.171	0.214	0.594
	440	440.10 ± 0.535	100.02	0.122	0.239	0.664
Interday assay	120	119.89 ± 0.447	99.91	0.373	0.200	0.555
	280	279.89 ± 0.610	99.96	0.217	0.273	0.757
	440	439.78 ± 0.811	99.95	0.363	0.363	1.007
**Method C**						
Intraday assay	20	20.00 ± 0.096	100.00	0.481	0.043	0.119
	40	40.01 ± 0.113	100.04	0.283	0.051	0.141
	60	59.98 ± 0.150	99.96	0.250	0.067	0.186
Interday assay	20	19.98 ± 0.174	99.89	0.872	0.078	0.216
	40	39.99 ± 0.149	99.98	0.373	0.067	0.185
	60	60.00 ± 0.244	100.00	0.406	0.109	0.303
**Method D**						
Intraday assay	40	40.02 ± 0.176	100.06	0.440	0.079	0.219
	100	100.05 ± 0.269	100.06	0.269	0.121	0.335
	160	160.02 ± 0.228	100.01	0.142	0.102	0.283
Interday assay	40	40.02 ± 0.367	100.06	0.918	0.164	0.456
	100	100.05 ± 0.420	100.06	0.420	0.188	0.521
	160	160.02 ± 0.509	100.01	0.318	0.228	0.632

^a^RSD, relative standard deviations (n = 5).

^b^SAE, standard analytical error.

^c^CL, confidence limit at 95% confidence level.

**Table 3.** Determination of enalapril maleate in pharmaceutical preparations by the proposed methods A, B, C, D and reference method (12).

**Formulations**	**Method A**		**Method B**		**Reference method**	
	**Recovery (%)**	**RSD (%)**	**Recovery (%)**	**RSD (%)**	**Recovery (%)**	**RSD (%)**
Enapril 2.5	100.03	1.159	100.16	1.014	99.92	1.206
	θ_L_ = 0.993	θ_U_ = 1.005	θ_L_ = 0.992	θ_U_ = 1.003		
	t = 0.505	F = 1.081	t = 1.158	F = 1.407		
Envas 2.5	99.93	1.314	99.98	1.162	99.81	1.383
	θ_L_ = 0.988	θ_U_ = 1.002	θ_L_ = 0.991	θ_U_ = 1.005		
	t = 0.490	F = 1.106	t = 0.706	F = 1.411		
Enace 2.5	99.93	0.997	99.98	0.996	99.83	1.273
	θ_L_ = 0.993	θ_U_ = 1.005	θ_L_ = 0.993	θ_U_ = 1.005		
	t = 0.450	F = 1.627	t = 0.698	F = 1.694		
	**Method C**		**Method D**			
Enapril 2.5	100.06	1.048	100.13	0.959	99.92	1.206
	θ_L_ = 0.993	θ_U_ = 1.005	θ_L_ = 0.985	θ_U_ = 1.011		
	t = 0.219	F = 1.321	t = 1.041	F = 1.578		
Envas 2.5	99.98	1.314	100.13	1.222	99.81	1.383
	θ_L_ = 0.991	θ_U_ = 1.006	θ_L_ = 0.990	θ_U_ = 1.004		
	t = 0.018	F = 1.023	t = 0.706	F = 1.272		
Enace 2.5	100.06	1.210	99.92	1.177	99.83	1.273
	θ_L_ = 0.996	θ_U_ = 1.009	θ_L_ = 0.993	θ_U_ = 1.006		
	t = 0.964	F = 1.101	t = 0.361	t = 0.361		

**Table 4.** Application of the proposed spectrophotometric methods to the determination of enalapril maleate in spiked human urine.

	**Amount added (μg/mL)**	**Amount found (μg/mL)**	**% Recovery**
Method A	10.0	9.83	98.30
	25.0	24.63	98.52
	40.0	39.56	98.91
	*χ̄*		98.58
	RSD		0.31
Method B	80.0	79.14	98.92
	320.0	319.25	99.76
	480.0	474.28	98.81
	*χ̄*		99.16
	RSD		0.52
Method C	20.0	19.45	97.25
	40.0	38.78	96.96
	100.0	99.52	99.52
	*χ̄*		97.91
	RSD		1.43
Method D	40.0	39.57	98.93
	80.0	79.83	99.78
	160.0	158.70	99.19
	*χ̄*		99.30
	RSD		0.44
